# Relative validity of the food frequency questionnaire used to assess dietary intake in the Leiden Longevity Study

**DOI:** 10.1186/1475-2891-12-75

**Published:** 2013-06-07

**Authors:** Martinette T Streppel, Jeanne HM de Vries, Saskia Meijboom, Marian Beekman, Anton JM de Craen, P Eline Slagboom, Edith JM Feskens

**Affiliations:** 1 Division of Human Nutrition, Wageningen University, Wageningen, The Netherlands; 2 Department of Molecular Epidemiology, Leiden University Medical Center, Leiden, The Netherlands; 3 Department of Gerontology and Geriatrics, Leiden University Medical Center, Leiden, The Netherlands; 4 Netherlands Consortium for Healthy Ageing, Leiden, The Netherlands

**Keywords:** Relative validity, Food frequency questionnaire, 24-hour recall

## Abstract

**Background:**

Invalid information on dietary intake may lead to false diet-disease associations. This study was conducted to examine the relative validity of the food frequency questionnaire (FFQ) used to assess dietary intake in the Leiden Longevity Study.

**Methods:**

A total of 128 men and women participating in the Leiden Longevity Study were included in the present validation study. The performance of the FFQ was evaluated using the mean of three 24-hour recalls as the reference method. Evaluation in estimating dietary intake at the group level was done by paired t-tests. The relative validity of the individual energy adjusted level of intake was assessed with correlation analyses (Pearson’s), with correction for measurement error.

**Results:**

On group level, the FFQ overestimated as well as underestimated absolute intake of various nutrients and foods. The Bland and Altman plot for total energy intake showed that the agreement between the FFQ and the 24-hour recalls was dependent of intake level. Pearson correlation coefficients ranged from 0.21 (alpha linolenic acid) to 0.78 (ethanol) for nutrients and from -0.02 (legumes, non-significant) to 0.78 (alcoholic beverages) for foods. Adjustment for energy intake slightly lowered the correlation coefficients for nutrients (mean coefficient: 0.48 versus 0.50), while adjustment for within-subject variation in the 24-h recalls resulted in higher correlation coefficients for both nutrients and foods (mean coefficient: 0.69 for nutrients and 0.65 for foods).

**Conclusions:**

For most nutrients and foods, the ability of the FFQ to rank subjects was acceptable to good.

## Background

Because they are able to rank subjects according to their intake and are relatively inexpensive, food frequency questionnaires (FFQs) are often used in epidemiological studies to assess usual dietary intake [1]. In a FFQ, subjects report the frequency of consumption and optionally portion sizes of a finite list of food items over a specific period of time in the recent past, for example the previous year or month. Differences in FFQ design characteristics, e.g. the number of food items, the inclusion of portion size questions, and mode of administration, can affect the validity of a FFQ [2]. Furthermore, the validity of the same FFQ may vary from population to population. Evaluation of a FFQ is important because invalid information on dietary intake may lead to false diet-disease associations. Therefore, validation studies should be performed to examine the degree to which the FFQ agrees with the subjects’ true intake [3]. Moreover, validation studies can be carried out to assess the level of measurement error associated with the FFQ [3].

Within the Leiden Longevity Study, a self-administered FFQ was developed to assess dietary intake. The Leiden Longevity Study (LLS) aims to identify heritable determinants explaining the familial differences in human longevity. In this study design, measures among offspring of nonagenarians siblings are compared to those among their partners that are considered as similarly aged controls from the general population. For potential determinants of healthy ageing we expect that nutrition may be a confounding factor, but dietary intake itself will also be investigated as potential determinant of healthy ageing. In the present study, we report on the relative validity of energy, nutrient and food intake estimated by the FFQ in the offspring of nonagenarians and the control population. We used multiple 24-hour recalls as the reference method.

## Methods

### Subjects

In the LLS, 420 families were recruited if at least two long-lived siblings were alive and fulfilled the age criterion of ≥89 years for men and ≥91 years for women [4]. As no proper controls exist for this age group, 1671 offspring of these nonagenarians, as a group of healthy agers prone to become long-lived, were included for further studies. This generation carries on average 50% of the genetic advantage of their long-lived parent and was shown to have a 35% lower mortality rate than their birth cohort [4]. In addition, 744 of their partners were included as population-based controls. By recruiting long-lived siblings and their offspring, the population was genetically enriched for longevity [4]. The partners of the offspring were included as the control population as they are likely to have the same environmental background, including dietary habits. Ethical approval was provided through the Medical Ethics Committee of the Leiden University Medical Center. Written informed consent was obtained from all participants.

Information on dietary habits and nutrient intake was only collected in the offspring of nonagenarians and their partners. 1630 Participants (Noffspring=1151, Ncontrol=479) completed a food frequency questionnaire (FFQ). Of these, 128 participants (Noffspring=62, Ncontrol=66) reported their 24-hour intake for three days.

### Methods of dietary assessment

Using a FFQ, participants reported the intake of foods consumed during the previous month. The FFQ was designed for the Dutch population and based on the VetExpress, a 104-item FFQ, valid for estimating the intake of energy, total fat, saturated (SFA), monounsaturated (MUFA), and polyunsaturated fatty acids (PUFA), and cholesterol in adults [5]. The VetExpress was updated and extended with vegetables, fruit, and foods for estimating the intake of specific PUFA’s, vitamins, minerals, and flavonoids. To identify relevant foods and food groups for this questionnaire, food consumption data of the Dutch National Food Survey of 1998 were used. Foods that contributed >0.1% to the intake of one of the nutrients of interest of adults were added in this survey. Thus, the FFQ is expected to include foods that cover the daily intake of each nutrient of food of interest for at least 90%. In a final step, foods were clustered to food items and extended with new foods on the market and foods to guarantee face validity. The FFQ was sent to each study participant, and after completing it, the participants returned the FFQ in an envelope free of postal charge. A dietician went through each FFQ to check for completeness. If necessary, she contacted the participants by telephone and obtained information on unclear or missing items. The FFQ also included questions on adherence to a special diet as well as questions about the use of dietary supplements.

Some of the offspring and their partners who completed the general questionnaire of the LLS were invited to the clinic for additional measurements at the Leiden University Medical Center. These measurements lasted a half day and couples were invited for the morning program or the afternoon program, which were slightly different due to practical reasons. The first 24-hour recall was performed in those participants who came to the clinic for the measurement in the morning program [N=128 (Noffspring=62, Ncontrol=66)]. A dietician asked the participants about their dietary intake of the previous day covering all foods and beverages consumed from waking up until the next morning. The dieticians received standardized training, using a formal protocol, to reduce the impact of the interview on the reporting process. For the two remaining recalls, the dietician contacted the participants by telephone within the next seven days. The 24-hour recalls were performed throughout the year and the days were chosen non-consecutively. They include a randomly assigned combination of days of the week with all days of the week represented (80% weekdays and 20% weekend days), for each individual.

The food data from both dietary assessment methods were converted into energy and nutrient intake by using the NEVO food composition database of 2006 [6]. Furthermore, foods were categorized into 24 major food groups. Age was calculated from date of birth and completion date of the FFQ. For subjects with missing information on the date of completing the FFQ, we used the median date of the other subjects.

### Assessment of additional information

Additional information was collected, including self-reported information on lifestyle (e.g. alcohol consumption and smoking habits). In the present study, alcohol use was defined as drinking at least 1 glass of alcoholic beverages per week and current smoking as smoking at least 1 cigarette per month. Information on medical history was collected from the participants’ general practitioners. Body mass index (BMI, kg/m^2^) was calculated using self-reported weight and height.

### Statistical analysis

Mean crude and energy-adjusted dietary intake (and SD) was calculated for each dietary assessment method. The performance of the FFQ in estimating dietary intake at the group level was determined by a paired t-test. Agreement between the two methods in assessing total energy intake was visualized by plotting the difference between the FFQ and the 24-hour recalls against the mean of the two methods [7]. In addition, we performed linear regression analysis with the difference between the FFQ and the 24-hour recalls as the outcome variable and the mean of the two methods as the predictor variable.

The relative validity of the individual energy adjusted level of intake was assessed with correlation analyses (Pearson’s), with correction for measurement error. To correct for attenuation due to within-subject variation in multiple 24-hour recalls (=reference method), the following formula was used:

$$r_{c}=r_{0}*{\left[1+\left(Sw^{2}/Sb^{2}\right)/n\right]}^{0.5}$$

where *r*_*c*_ = corrected/de-attenuated correlation coefficient; *r*_*0*_ = uncorrected/attenuated correlation between the FFQ and the 24-hour recalls; *Sw*^*2*^ = within-subject variance of the multiple 24-hour recalls*; Sb*^*2*^ = between-subject variance of the 24-hour recalls; and *n* = number of repeated measures of the 24-hour recalls.

The ratio of energy intake (EI) to basal metabolic rate (BMR) was calculated to evaluate underreporting. A cut-off value for EI to BMR ratio to identify underreporting was set [8-10]. We assumed a within-subject variation in energy intake of 23%, a within-subject variation in estimated BMR of 8.5%, a physical activity level (PAL) of 1.55, and a between-subject variation in PAL of 15%. BMR was predicted from the standard equation from Henry *et al.*[11].

## Results

Table 1 shows some background characteristics of study participants who provided FFQ (N=1630) and both FFQ and 24-hour recall data (N=128). The men included in the present validation study (N=61, 48%) were older, had a higher BMI and more often hypertension and diabetes as compared to the women. In contrast, the women more often used dietary supplements. Aside from the percentage of offspring and subjects with a medical condition, the characteristics of the subjects in the present validation study were comparable to all participants of the LLS study that provided FFQ data.

**Table 1:** Baseline characteristics of participants of the Leiden Longevity Study providing FFQ data

		***Subjects with FFQ and 24h recall data***		
**Variable**	**All subjects**	**All**	**Men**	**Women**
N	1630	128	62	66
Offspring, n (%)	1151 (71)	62 (48)	30 (48)	32 (48)
Age, years	62 ± 7	62 ± 6	64 ± 6	61 ± 6
Males, n (%)	709 (44)	62 (48)	61 (100)	0 (0)
Body mass index, kg/m^2^	25.3 ± 3.5^a^	25.7 ± 3.4^e^	26.0 ± 2.7	25.3 ± 4.0
Energy intake to basal metabolic rate ratio	1.32^a^	1.27^e^	1.24	1.29
Alcohol use, n (%)	1133 (72)^b^	95 (74)	51 (82)	44 (67)
Supplement use, n (%)	573 (35)	45 (35)	17 (27)	28 (42)
Prescribed diet, n (%)	189 (12)	15 (12)	7 (11)	8 (12)
Current cigarette smoking, n (%)	193 (12)^c^	13 (10)^f^	6 (10)	7 (11)
Medical condition, n (%)	476 (29)^d^	50 (39)^g^	26 (42)	24 (36)
*Myocardial infarction, n (%)*	*36 (2)*	*0 (0)*	*0 (0)*	*0 (0)*
*Cerebrovascular accident, n (%)*	*40 (3)*	*2 (2)*	*1 (2)*	*1 (2)*
*Hypertension, n (%)*	*332 (23)*	*40 (36)*	*22 (41)*	*18 (32)*
*Diabetes mellitus, n (%)*	*60 (4)*	*9 (8)*	*7 (13)*	*2 (4)*
*Cancer, n (%)*	*122 (7)*	*10 (8)*	*5 (8)*	*5 (8)*

a = Data on BMI and EI to BMR ratio were missing for 48 subjects; b= Data on alcohol use were missing for 47 subjects; c = Data on current smoking were missing for 49 subjects; d = Data on MI, cerebrovascular accident, hypertension and diabetes were missing for, respectively, 182, 183, 209 and 198 subjects; e = Data on BMI and EI to BMR ratio were missing for 2 subjects; f = Data on current smoking were missing for one subject; g = Data on cerebrovascular accident, hypertension and diabetes were missing for, respectively, 15, 17 and 20 subjects.

### Energy and nutrient intake

In Table 2, the mean energy and nutrient intake for the FFQ and 24-hour recalls (N=128) is presented. For

**Table 2:** Difference in energy and nutrient intakes and Pearson’s correlation coefficients between the FFQ and 24-hour recalls (N=128)

	**FFQ**		**24-hour recall**			**Pearson's correlation coefficients**		
**Nutrient**	**Mean**	**S.d.**	**Mean**	**S.d.**	***p-value***^***a***^	**Crude**^**a**^	**Adj**^**a,b**^	**Adj, deatt**^**a,b,c**^
Energy (kcal)	1914	542	1891	427	0.95	0.55	-	0.65
Total protein (g)	72	22	76	19	0.02	0.51	0.50	0.61
Vegetable protein (g)	28	9	28	8	0.64	0.67	0.63	0.86
Animal protein (g)	44	16	48	16	0.002	0.49	0.54	0.60
Total fat (g)	76	28	70	21	0.05	0.39	0.43	0.50
Saturated fatty acids (g)	28	12	26	9	0.38	0.44	0.57	0.55
Monounsaturated fatty acids (g)	26	9	23	8	0.02	0.33	0.33	0.46
Polyunsaturated fatty acids (g)	16	7	14	5	<.0001	0.44	0.44	0.57
Trans fatty acids (g)	2	1	2	1	0.004	0.42	0.44	0.61
Linoleic acid (g)	13	6	11	5	0.0002	0.43	0.40	0.56
Alpha linolenic acid (g)	1.32	0.80	0.86	0.45	<.0001	0.21	0.21	0.29
EPA (g)	0.06	0.06	0.09	0.20	0.21	0.39	0.40	0.48
DHA (g)	0.09	0.09	0.14	0.26	0.15	0.36	0.38	0.50
Cholesterol (mg)	194	79	203	90	0.44	0.42	0.41	0.75
Total carbohydrates (g)	206	58	209	55	0.49	0.70	0.66	0.80
Mono- disaccharides (g)	102	34	102	36	0.85	0.61	0.50	0.69
Polysaccharides (g)	104	34	106	29	0.11	0.70	0.68	0.87
Dietary fiber (g)	23	7	21	7	0.01	0.66	0.59	0.82
Ethanol (g)	16	14	17	16	0.68	0.78	0.78	0.95
Calcium (mg)	982	489	986	316	0.21	0.56	0.59	0.67
Vitamin B1 (mg)	1.23	0.36	1.15	0.43	0.00	0.58	0.45	0.86
Vitamin B2 (mg)	1.58	0.64	1.51	0.47	0.26	0.58	0.52	0.69
Vitamin B6 (mg)	1.65	0.49	1.69	0.48	0.33	0.52	0.35	0.67
Vitamin B12 (μg)	4.43	2.23	4.52	4.20	0.28	0.43	0.41	0.72
Vitamin D (μg)	3.97	1.87	4.05	2.40	0.90	0.49	0.47	0.74
Vitamin E (mg)	13	5	11	5	0.001	0.38	0.36	0.46
Vitamin C (mg)	94	39	99	46	0.79	0.51	0.49	0.68
Lycopene (μg)	2373	3900	2025	3718	<.0001	0.46	0.48	1.26
Retinol activity equivalent (μg)	1023	618	771	499	<.0001	0.54	0.46	0.87
Folic acid equivalent (μg)	196	65	184	55	0.03	0.53	0.35	0.87

a = Based on log-transformed values; b = Correlation coefficients were adjusted for energy intake; energy-adjusted intakes were calculated using the residual method; c = Correlation coefficients were de-attenuated (corrected for within-person variation [derived from crude, untransformed data] in the 24-hour recalls).

**Figure 1:**
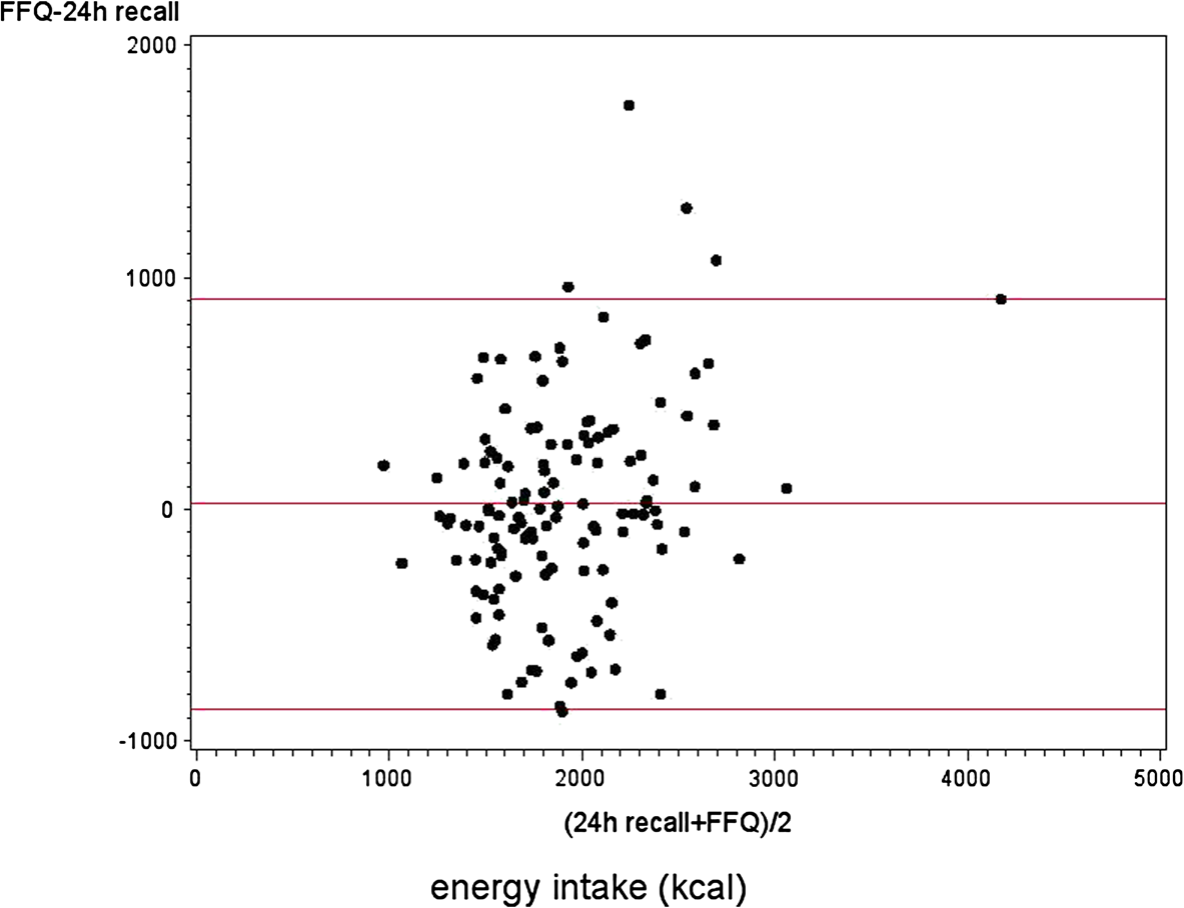
**Bland-Altman plot of total energy intake.** Differences in the daily intake of total energy estimated with 24-hour recalls and a food frequency questionnaire, plotted against the mean daily intake estimated by the two methods (N=128). Mean difference and 95% limits of agreement (1.96 × SD of mean difference) are included.

 MUFA, PUFA, and dietary fiber, mean intake as estimated by the FFQ was significantly higher than the intake estimated by the 24-hour recalls. In contrast, mean intake of total protein, animal protein, trans fatty acids and polysaccharides was lower when estimated by the FFQ as compared to the 24-hour recalls. In addition, the estimated mean intake of vitamin B1, E, lycopene, retinol activity equivalents (RAE) and folic acid equivalents (FAE) by the FFQ was significantly higher than by the 24-hour recalls. Figure 1 shows the Bland-Altman plot for total energy intake. We observed an increasing difference between the FFQ and 24-hour recalls with increasing mean values of total energy intake (intercept: -554.04, p-value: 0.002; slope: 0.299 per kcal increase, p-value: 0.001).

The correlation coefficients between the FFQ and 24-hour recalls ranged from 0.21 for alpha linolenic acid (ALA) intake to 0.78 for ethanol intake (mean: 0.50; Table 2). Adjustment for total energy intake resulted in slightly lower correlation coefficients for most nutrients [mean: 0.48; range 0.21 (ALA) – 0.78 (ethanol)]. Adjustment for the within-subject variation of the repeated 24-hour recalls resulted in de-attenuated and adjusted correlation coefficients ranging from 0.29 for

 ALA to 1.26 for lycopene (mean: 0.69). The effect of de-attenuation was most pronounced for dietary cholesterol, vitamin B12 and lycopene, due to the relatively large day-to-day variation.

The average ratio of EI to BMR was 1.32 for the total LLS population (N=1630) and 1.27 for the subjects included in the present study (N=128; Table 1). These ratios are below the estimated cut-off values (1.54 for the total LLS population and 1.50 for the subjects in the present study) Linear regression analyses showed that there was a significant inverse association between the EI to BMR ratio and BMI (slope: -0.029 per kg/m^2^ increase, p-value: <.0001). On an individual level, 30% of the total LLS population that provided FFQ data had an EI to BMR ratio below the individual cut-off value of 1.10. This percentage was a bit higher in the subjects selected for the present study (34%).

### Food consumption

The average consumption of foods for the FFQ and 24-hour recalls (N=128) is presented in Table 3. For most food groups, there was a significant difference in average consumption estimated by the FFQ and the consumption estimated by the 24-hour recalls. The average consumption of cereal products, savory sandwich fillings, soya and vegetarian products, nuts and seeds, legumes, and sugar and sweets was higher when estimated by the FFQ as compared to the 24-hour recalls. In contrast, the average consumption of non-alcoholic beverages, bread, eggs, composite dishes, soups, fish, and meat products were lower when estimated by the FFQ.

For non-alcoholic beverages, legumes, and composite dishes, we observed no correlation between the intake estimated by the FFQ and the 24-hour recalls (Table 3). For the other food groups, the correlation coefficients ranged from 0.24 for vegetable consumption to 0.79 for bread consumption (mean: 0.49). Adjustment for total energy intake did not change these correlation coefficients. The de-attenuated and adjusted correlation coefficients ranged from 0.40 for nuts, seeds and snacks to 0.98 for meat, meat products and poultry (mean: 0.65). The effect of de-attenuation was most pronounced for potatoes, vegetables, nuts and seeds, soups, and meat products.

**Table 3:** Difference in food consumption and Pearson’s correlation coefficients between the FFQ and 24-hour recalls (N=128)

	**FFQ**		**24-hour recall**			**Pearson's correlation coefficients**		
**Food group**	**Mean**	**S.d.**	**Mean**	**S.d.**	***p-value***^***a***^	**Crude**^**a**^	**Adj**^**a,b**^	**Adj, deatt**^**a,b,c**^
Potatoes (g)	90	57	104	61	0.88	0.25	0.21	0.50
Non-alcoholic beverages (g)	790	311	1631	601	<.0001	0.14^d^	0.17^d^	0.15^d^
Bread (g)	126	55	133	54	0.002	0.79	0.71	0.93
Eggs (g)	12	10	13	17	<.0001	0.46	0.46	-
Fruit (g)	184	124	163	118	0.28	0.50	0.51	0.62
Pastry, cake and biscuits (g)	36	23	41	33	0.13	0.63	0.65	0.86
Cereal products and binding agents (g)	43	35	26	33	<.0001	0.41	0.40	-
Vegetables (g)	136	72	141	76	0.80	0.24	0.23	0.41
Savory sandwich fillings (g)	2	5	2	5	0.01	0.38	0.39	0.45
Cheese (g)	36	38	30	20	0.38	0.46	0.51	0.61
Milk and milk products (g)	311	221	328	223	0.27	0.69	0.68	0.75
Soya products and vegetarian products (g)	10	33	6	22	0.01	0.50	0.51	0.66
Nuts, seeds and snacks (g)	32	26	18	24	<.0001	0.24	0.25	0.40
Legumes (g)	8	11	2	9	<.0001	−0.02^d^	−0.01^d^	-
Composite dishes (g)	14	15	28	54	0.0001	0.10^d^	0.10^d^	-
Soups (g)	53	62	52	76	<.0001	0.31	0.30	0.66
Sugar, confectionary, sweet fillings and sweet sauces (g)	28	24	25	23	0.02	0.67	0.66	0.81
Fats, oils and savory sauces (g)	40	20	40	27	0.32	0.53	0.54	0.77
Fish (g)	14	14	19	40	<.0001	0.37	0.37	0.46
Meat, meat products and poultry (g)	75	34	94	48	0.02	0.56	0.56	0.98
Alcoholic beverages (g)	196	243	217	287	0.05	0.78	0.75	0.88

a = Based on log-transformed values; b = Correlation coefficients were adjusted for energy intake; energy-adjusted intakes were calculated using the residual method; c = Correlation coefficients were de-attenuated (corrected for within-person variation [derived from crude, untransformed data] in the 24-hour recalls); d = Correlation coefficients are not significantly different from zero.

## Discussion

The objective of the present study was to examine the relative validity of the FFQ used to assess dietary intake in the Leiden Longevity Study (LLS). The FFQ overestimated as well as underestimated the absolute intake of various nutrients and foods. We observed that the agreement between the two dietary assessment methods in estimating total energy intake was dependent of the intake level. Pearson correlation coefficients ranged from 0.21 (ALA) to 0.78 (ethanol) for nutrients and from -0.02 (NS, legumes) to 0.79 (alcoholic beverages) for foods. Adjustment for total energy intake slightly lowered the correlation coefficients for nutrients, while adjustment for within-subject variation in the 24-hour recalls resulted in higher correlation coefficients for both nutrients and foods. The subjects included in the present validation study were a representative

 sample of the total LLS population that provided FFQ data with respect to age, gender, BMI, and lifestyle factors, like smoking habits, alcohol use and being on a prescribed diet. Therefore, the findings of the validation study can be extrapolated to the total LLS population.

Although the estimated mean energy intake did not differ between the FFQ and 24-hour recalls, we did observe that the agreement worsened as total energy intake increased. Siebelink *et al.* assessed how accurately participants report their energy intake by a comparable FFQ; they compared reported energy intake with actual energy intake needed to maintain a stable body weight during controlled dietary trials [12]. Just like the present study, Siebelink *et al.* observed a general trend of under-reporting of energy intake at lower intakes and over-reporting at higher intakes [12]. These results suggest the FFQ is able to estimate total energy intake on a group level, but not on an individual level.

To study diet-disease relationships, ranking of subjects according to their dietary intake is more important than estimating their absolute intake level. Therefore, we examined the relative validity of the FFQ as compared to the 24-hour recalls by calculating Pearson’s correlation coefficients. Adjustment for measurement error in the 24-hour recalls resulted in higher correlation coefficients. For most nutrients and foods, we observed acceptable to (very) good correlations between the FFQ and 24-hour recalls. For the macronutrients, vitamins and minerals, the correlations were comparable to other FFQs [2,3,13,14]. With regard to SFA, MUFA, and PUFA, the correlations were lower than found for the original VetExpress questionnaire [5]. However, the correlations observed by Feunekes *et al.*[5] were probably overestimated because they used a dietary history as reference method which is based on the same principle as the FFQ. Although the FFQ in the LLS was designed to assess habitual ALA intake, we observed a poor correlation (*r*<0.30) between the FFQ and 24-hour recalls, even after adjustment for measurement error in the 24-hour recalls. In the Netherlands, average ALA intake is about 1.7 g per day in men and 1.2 gram per day in women [15]. This is lower than the intake estimated using the mean of three 24-hour recalls in the present validation study. As ALA occurs in foods that are consumed infrequently, our reference method may not be suitable to validate the FFQ with regard to ALA intake. Other FFQ validation studies used biomarkers or weighted food records that assess the intake >7 days. In these studies, the summarized correlation for ALA was poor for studies that used biomarkers as reference and acceptable for studies that used weighted food records as reference [16].

In addition to nutrients, we also examined the relative validity of the FFQ in estimating habitual consumption of foods. The crude correlation between the FFQ and 24-hour recalls was low for potatoes, vegetables, and nuts and seeds. These correlations were also lower than those observed for the Dutch EPIC questionnaire [17]. However, adjustment for measurement error in the 24-hour recalls resulted in acceptable correlations (*r*>0.40) for these foods. For non-alcoholic beverages, legumes, and composite dishes, we observed no correlation between the FFQ and the 24-hour recalls. In the Netherlands, legumes are consumed infrequently and using the mean of three 24-hour recalls as a reference method may not be not appropriate. With regard to non-alcoholic beverages, our FFQ seems to be unsuitable to rank subjects according to their intake. This is in contrast with the correlation for the Dutch EPIC questionnaire, which was good (*r*=0.67 for men and 0.49 for women). Our FFQ was not designed to estimate the intake of liquids and –contrary to the EPIC questionnaire– did not include specific questions about tap water. This may explain the large difference between the intake of non-alcoholic beverages estimated by the 24-hour recalls and the intake estimated by the FFQ found in the present study.

To evaluate underreporting, we calculated EI to BMR ratios and compared them to predetermined cut-off values [8-10]. The calculated EI to BMR ratios indicated underreporting of energy intake on group level. Previous studies have suggested that the probability of underreporting increases with increasing BMI [18]. In the present validation study, we indeed observed an inverse association between the EI to BMR ratio and BMI, indicating that the magnitude of underreporting increases with increasing BMI. This may affect diet-disease relationships. On individual level, ~30% of the subjects had an EI to BMR ratio below the cut-off value. According to Black [9], data on physical activity are needed to identify diet reports of poor validity. Unfortunately, this information was not available in the present study. To set the cut-off values for underreporting, we assumed a PAL of 1.55, which is the estimated average for a sedentary lifestyle. When the actual PAL is higher, the magnitude of underreporting on group and individual level in the present study is higher. However, as a FFQ is in general not suitable to estimate an individual’s absolute energy intake, and one should be careful when excluding subjects with an EI to BMR ratio below the cut-off value.

A vital component in validating a FFQ is the selection of the appropriate reference method. We used 24-hour recalls as the reference method. 24-Hour recalls are suitable to assess dietary intake on group levels but repeated recalls are needed to estimate usual intake, i.e. capture daily variation at an individual level [1]. The mean of three 24-hour recalls, as was used in the present study, may not be sufficient to capture the daily variation of foods that are consumed infrequently. As a result, the reference method will perform worse in estimating usual consumption of those foods –and thus the intake of specific nutrients from those foods– than a FFQ. In general, using another dietary assessment method as reference has its limitations. In their literature review, Poslusna *et al.* found that misreporting also occurs when using 24-hour recalls or food records to estimate dietary intake [19]. Thus, measurement errors –both systematic as well as random errors– exist in every dietary assessment method. For validation, especially the random errors need to be uncorrelated; however, this is usually not the case when using another dietary assessment method as reference. A FFQ and a 24-hour recall share common errors as they are both methods based on memory and the same food composition table is used to convert the foods to energy and nutrient intake. Random variation in the 24-hour recalls and the correlated errors in the repeated recalls may underestimate the correlation between the intake assessed with the FFQ and the true intake. On the other hand, correlated errors between the FFQ and the 24-hour recalls may overestimate this correlation. Nowadays, dietary biomarkers, i.e. biochemical indicators of dietary intake or nutritional status; indexes of nutrient metabolism; or markers of the biological consequences of dietary intake [20], are more often being used as reference method as they are an objective measure of dietary intake and are independent of all the biases and errors associated with dietary assessment methods [21,22]. Unfortunately, no data on dietary biomarkers were available in this validation study.

## Conclusions

For most nutrient and foods, the ability of the FFQ to rank subjects according to their dietary intake was acceptable to good. The FFQ developed to assess dietary intake in the LLS can be used to study diet-disease relationships.

## Abbreviations

FFQ: Food frequency questionnaire; LLS: Leiden longevity study; SFA: Saturated fatty acids; MUFA: Monounsaturated fatty acids; PUFA: Polyunsaturated fatty acids; BMI: Body mass index; EI: Energy intake; BMR: Basic metabolic rate; PAL: Physical activity level; RAE: Retinol activity equivalents; FAE: Folic acid equivalents; ALA: Alpha linolenic acid; NS: Non-significant

## Competing interests

The authors declare they have no competing interests.

## Authors’ contributions

The contributions of the authors were as follows: MS participated in the manuscript conception, statistical analyses, data interpretation, manuscript writing and revising; JdV and SM contributed to the design of the study, development of the FFQ and data collection, and participated in data interpretation and review; MB, AdC, and ES contributed to the conception of the study, data interpretation and review; EF participated in the conception and design of the study, manuscript conception, data interpretation, writing and review. All authors contributed to the critical revision of the manuscript. All authors read and approved the final manuscript.
